# Retraction: Analysis of the role and robustness of artificial intelligence in commodity image recognition under deep learning neural network

**DOI:** 10.1371/journal.pone.0311323

**Published:** 2024-09-26

**Authors:** The PLOS ONE Editors

The *PLOS ONE* Editors retract this article [1] because it was identified as one of a series of submissions for which we have concerns about peer review integrity and similarities across articles. These concerns call into question the validity and provenance of the reported results. We regret that the issues were not identified prior to the article’s publication.

The corresponding author responded to correspondence about this matter, but their comments did not resolve the concerns.

RC and YL did not agree with the retraction. MW either did not respond directly or could not be reached.
